# Food 3D Printing Equipment and Innovation: Precision Meets Edibility

**DOI:** 10.3390/foods14122066

**Published:** 2025-06-11

**Authors:** Shuailei Xiao, Junhao Yang, Ye Bi, Yi Li, Yanwu Cao, Mengdi Zhou, Guibing Pang, Xiuping Dong, Qiang Tong

**Affiliations:** 1 College of Mechanical Engineering and Automation, Dalian Polytechnic University, Dalian 116034, China; xiaoshuailei@outlook.com (S.X.); by990816@163.com (Y.B.); liyi.work.dlpu@outlook.com (Y.L.); 15556062673@163.com (Y.C.); pangguibingsx@163.com (G.P.); 2 SKL of Marine Food Processing & Safety Control, National Engineering Research Center of Seafood, Dalian 116034, China; dxiuping@163.com; 3 Academy of Food Interdisciplinary Science, Dalian Polytechnic University, Dalian 116034, China; zhoumengdididi@163.com; 4 Liaoning Province Collaborative Innovation Center for Marine Food Deep Processing, Dalian Technology Innovation Center for Chinese Prepared Food, Dalian Polytechnic University, Dalian 116034, China

**Keywords:** 3D printing, additive manufacturing, food processing, consumers, protein-based ink

## Abstract

Food 3D printing technology holds significant potential for personalized nutrition and the customization of 3D structures with specific shapes and textures. This emerging technology is driving significant innovations in food processing and manufacturing. This article reviews the current research and development opportunities in food 3D printing, with a focus on various types of 3D food printing technologies. We offer novel insights into the behavioral characteristics of various 3D printing techniques during the printing process, while also highlighting that the successful development of 3D-printed food products relies on four critical factors: (1) adaptability, (2) printability, (3) food safety, and (4) consumer acceptance. Special emphasis is placed on the application and potential challenges of 3D printing technology, aiming to foster technological breakthroughs and industrial applications, ultimately offering consumers safer, smarter, and more personalized food choices.

## 1. Introduction

The core of 3D printing, or additive manufacturing, lies in its ability to perform customized processing based on digital models, which simplifies the design and production processes while lowering manufacturing barriers. Hull pioneered stereolithography (SLA), a technique that uses ultraviolet lasers to solidify layers of photosensitive resins and construct three-dimensional objects, step by step, for complex parts [1]. After over 30 years of development, 3D printing technology has evolved from a proof of concept into a mature industry, continuing to advance rapidly [2,3]. Printing materials have evolved from early polymers to include metals, ceramics, sand, biological tissues, and more [4,5,6]. Printing accuracy has advanced from the millimeter scale to the current micron scale, with significant progress also made in food printing [7]. In the food industry, 3D printed food offers significant advantages in customizing meals for diverse groups, such as the elderly, children, and astronauts, reducing raw material waste, and enabling the creation of food with complex geometric shapes and internal porous structures [8]. As food 3D printing technology continues to advance, the range of printable materials is steadily expanding, including chocolate, cheese, cereals, fruits and vegetables, icing sugar, and minced meat [9,10,11,12,13,14]. In recent years, researchers have experimented with innovative ingredients, such as algae, plant proteins, and meat substitutes, to create predefined food structures [15,16,17,18].

**Figure 1:**
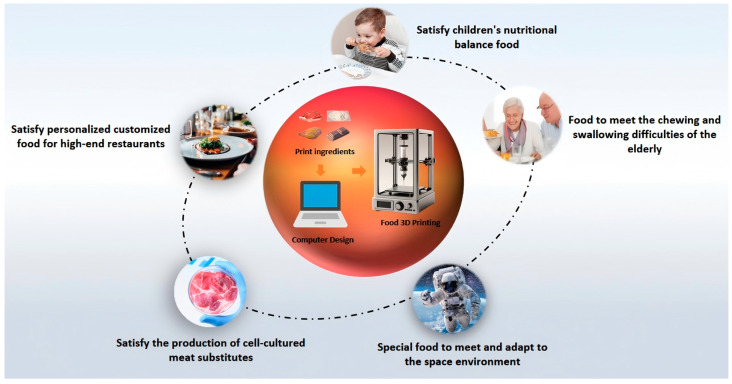
Food 3D printing application fields.

Currently, 3D food printing technology has garnered significant attention and has been extensively explored in various fields, including children’s food, elderly food, astronaut food, meat alternatives, and personalized customized food for restaurants, as detailed in Figure 1 [19]. Despite its widespread application in the food industry, there remains considerable room for improvement in this technology. Further exploration of new materials, enhancement of food safety and public awareness, and the development of nutritionally balanced and palatable 3D-printed foods are essential for their broader adoption.

Three-dimensional printing is being extensively researched in the field of food. However, existing studies primarily focus on printing processes and material development, while systematic research on critical issues such as types of 3D food printing technologies, food safety, and consumer acceptance remains relatively scarce. These subsequent evaluation metrics serve as important benchmarks for assessing the commercial viability of 3D-printed foods. The aim of this review paper is to systematically organize the current research status of 3D food printing technologies, with a particular emphasis on analyzing different 3D printing techniques and their applications in various food products. Additionally, it provides recommendations and offers critical insights into the trends and challenges facing 3D food printing.

## 2. Traditional Food 3D Printing Technology Types

The application of 3D printing in the food sector must take into account the unique properties of food materials and their specific processing requirements. Based on different manufacturing principles, a variety of process technologies suitable for 3D food printing have been developed. Table 1 systematically summarizes common 3D food printing technologies, categorizing them according to their underlying manufacturing principles while providing a detailed comparison of their process characteristics, applicable material ranges, advantages, and limitations.

## 3. Emerging Food 3D Printing Technology Applications

With the continuous innovation and application of 3D printing technology in the food sector, emerging techniques such as multi-axis, coaxial, laser, microwave, ultrasonic, and microfluidic printing have been developed, in addition to the traditional 3D food printing methods summarized in Table 1. These novel technologies offer expanded possibilities for 3D food printing, enabling the design and fabrication of more complex and intricate food structures.

### 3.1. Multi-Axis 3D Printing

Currently, the most common method in 3D food printing is single-axis extrusion, which involves depositing flowable materials through a single nozzle for shaping [27]. However, this technique suffers from relatively slow printing speeds, particularly when scaling up or achieving high-resolution prints, thereby limiting its efficiency [28]. Additionally, the single-nozzle design restricts the capability for multi-material co-printing, making it challenging to meet the demands of complex food or multifunctional structure fabrication. In contrast, multi-axis printing systems, equipped with multiple independent print heads, enable precise deposition and shaping of food materials, allowing for the creation of more intricate three-dimensional structures and multi-material prints. This capability facilitates the customization of both external shapes and internal architectures of food products [29,30,31].

**Figure 2:**
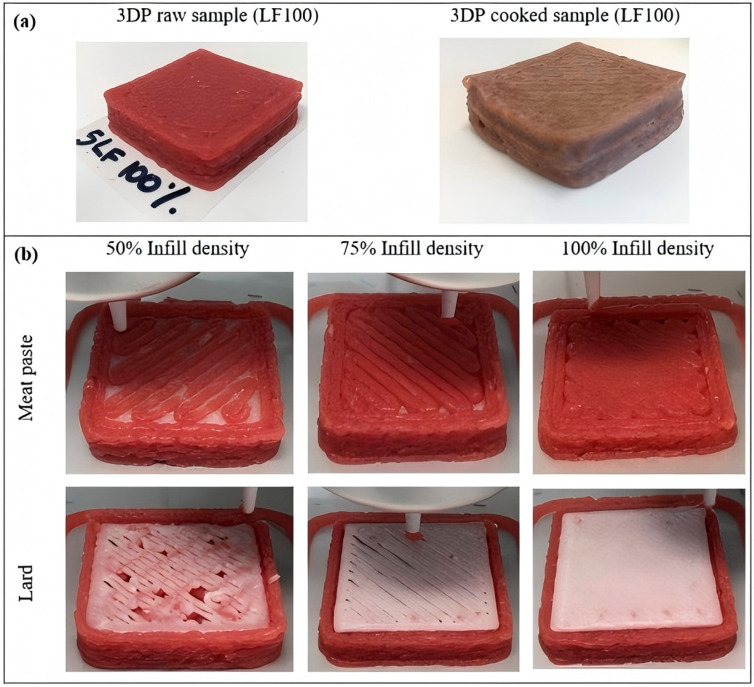
Three-dimensional printed composite multi-layer meat models [33]: (**a**) Raw and cooked samples (LF100: 1 layer of lard, 100% infill density), (**b**) filament stream at different infill densities (50%, 75%, and 100%) for meat paste (2 mm nozzle diameter) and lard (1 mm nozzle diameter).

**Figure 3:**
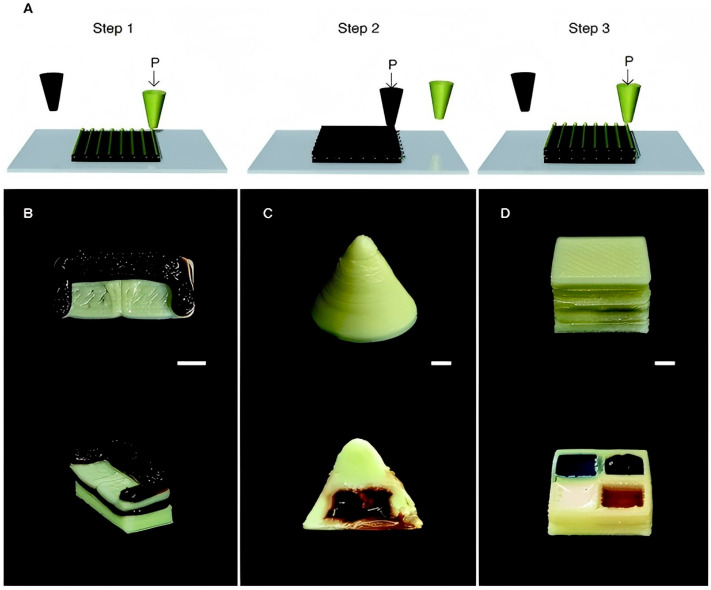
Three-dimensional printed multi-food models [38]: (**A**) Schematic illustration of multi-material DIW three-dimensional printing. (**B**) Three-dimensional structure of a couch printed with milk and chocolate inks at different layers. (**C**) Three-dimensional printed cone containing liquid chocolate syrup as an internal filling. (**D**) Three-dimensional printed cube with four compartments containing liquid blueberry syrup, liquid chocolate syrup, milk cream, and maple syrup as internal fillings (all scale bar: 5 mm).

Beef has long been one of the primary sources of high-quality protein for humans and ranks as the third most popular meat globally [32]. However, due to the poor printability of pure beef, most researchers have focused on modifying it to enhance its suitability for 3D printing [33,34]. Research indicates that consumers prefer beef with intramuscular fat deposition (i.e., marbling) when consuming beef products, considering tenderness a crucial factor, and are willing to pay a premium for more tender beef products. Dick et al. utilized a dual-nozzle extrusion-based 3D printing technology to investigate the effects of varying fill densities (50%, 75%, and 100%) and fat content (0, 1, 2, and 3 layers of fat within the structure) on the physical properties and texture of lean meat–lard composite layers during post-processing using sous-vide cooking, as illustrated in Figure 2 [35]. The results indicated that fill density was positively correlated with moisture retention, hardness, and chewiness, while negatively correlated with shrinkage and cohesiveness. However, fill density had no significant impact on fat retention. The 3D-printed objects successfully maintained their internal and external structures after cooking, but the distribution of intramuscular fat could not be regulated through fat content. In a recent study, Park et al. employed multi-material 3D printing to explore the regulation of intramuscular fat distribution by adjusting fat content, aiming to meet consumers’ varying preferences for marbled meat [36]. The results demonstrated that using a mixture of meat and fat during the printing process ensured shape stability, while the regional distribution of intramuscular fat within the meat could be controlled by modulating the fat content in the mixture. Furthermore, chocolate is one of the most popular materials in food printing due to its melt-extrusion capability and widespread appeal in the high-end food industry [37]. However, chocolate is a challenging medium to process because of its complex composition, primarily consisting of a mixture of cocoa, milk solids, and milk fat. Lee and Karyappa developed a direct ink writing (DIW) multi-material 3D printing method for dairy products at room temperature by modifying the rheological properties of the printing ink. Using a 70 w/w% milk-based ink, they successfully 3D printed intricate food structures, as shown in Figure 3 [38]. In the future, with continuous advancements in this technology, we can expect to see more astonishing 3D-printed delicacies on our dining tables.

### 3.2. Coaxial 3D Printing

**Figure 4:**
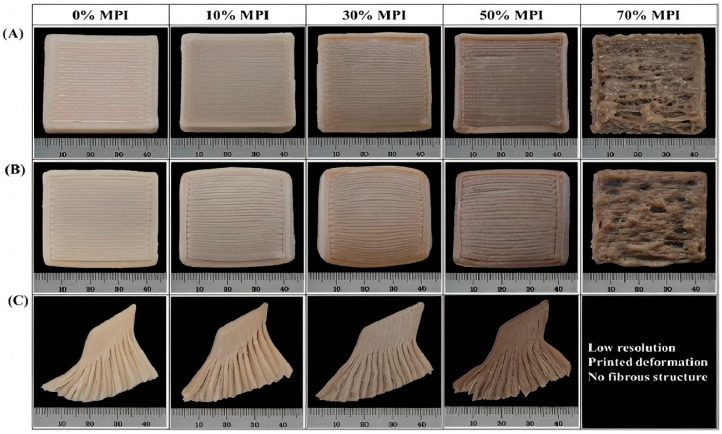
Coaxial 3D printing characteristics of CS incorporated with different contents of MPI gel [41]. (**A**) Printed raw surimi; (**B**) printed surimi after post-processing; (**C**) fibrous structure after post-processing.

**Figure 5:**
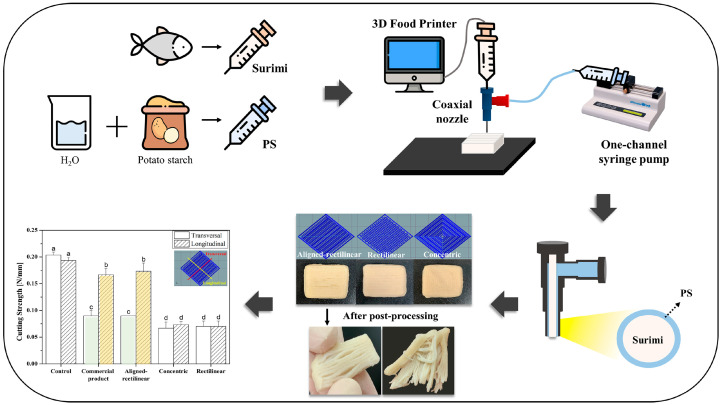
Graphical abstract of surimi-based imitation crab meat using coaxial extrusion 3D food printing [42].

Coaxial 3D printing technology features a dual-axis design, where the print head is equipped with separate output channels for both the food material and the support material, enabling their simultaneous extrusion. Since both the food material and the support material are precisely extruded concurrently, this method allows for the construction of more intricate internal cavities, hollow structures, or embedded architectures during the printing process. As individuals age, functional abilities gradually decline, leading to issues such as diminished taste perception, chewing difficulties, and swallowing disorders among the elderly [39]. China, as one of the most populous countries in the world, has a population of 264.02 million aged 60 and above and 190.64 million aged 65 and above, accounting for approximately 13.50% of the total population [40]. Chao et al. modified the texture of chicken surimi (CS) by incorporating yellow mealworm protein isolate (MPI) and successfully generated fiber-structured elderly food using coaxial 3D printing technology [41]. As the MPI content increased, the G’ value significantly decreased, indicating a reduction in the viscoelasticity of the surimi paste. After post-processing, the printed surimi exhibited improved water-holding capacity (WHC) and reduced cooking loss. A notable decrease in hardness was observed with increasing MPI content, which was associated with changes in the microstructure of the surimi. Meanwhile, the mixture of MPI (50%) and CS represented the maximum printable content and demonstrated a lower hardness value (4.13 × 10^4^ ± 0.61 N/m^2^), meeting the Stage 2 requirements of the Universal Design Food (UDF) guidelines. The coaxial 3D printing characteristics of CS gels with varying MPI contents are illustrated in Figure 4. Furthermore, Kim et al. employed coaxial extrusion 3D printing technology to uniformly coat a layer of potato starch (PS) solution on the surface of fish paste, successfully constructing products with imitation crab meat filament structures [42]. During the experimental process, researchers utilized rheometers to determine the rheological properties of PS solutions and fish paste prior to printing. Results demonstrated that with increasing PS concentration, the complex viscosity (η*), storage modulus (G′), and loss modulus (G″) all significantly increased, indicating that the constructed 3D printed structures possessed excellent mechanical strength. Further investigation into the effects of different processing conditions on printing performance within PS concentrations ranging from 0 to 15% (mass fraction) revealed that at a PS concentration of 12%, the printed fish paste exhibited optimal performance in terms of forming precision, water-holding capacity, and cooking loss. This research not only effectively achieved the fibrous structure reconstruction of fish paste products but also provided feasible solutions to key technological challenges in food 3D printing. The surimi-based crab meat analog produced using coaxial extrusion 3D food printing is illustrated in Figure 5.

**Figure 6:**
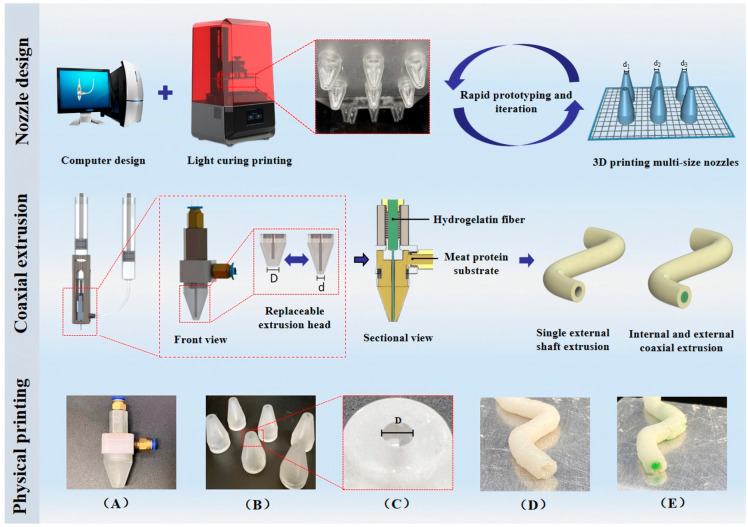
Coaxial extrusion principle and physical picture. (**A**) Coaxial nozzle. (**B**) Three-dimensional printing multi-size external axis nozzle. (**C**) Partial view of replaceable outer shaft diameter. (**D**) Single external shaft extrusion. (**E**) Internal and external coaxial extrusion.

Due to the simultaneous extrusion of materials through the inner and outer axes, ensuring speed compatibility between the two is crucial to avoid common 3D printing defects such as delamination and deformation. Currently, most researchers address this issue by modifying material properties or adjusting 3D printing parameters to regulate material flow rate and pressure [41,43]. To further enhance the dimensional accuracy and surface quality of printed objects, our team has designed a material extrusion system capable of supporting different diameters and flow rates, catering to the requirements of various food formulations. Additionally, we utilized 3D printing technology to achieve rapid prototyping and iterative optimization of nozzles, making nozzle design more flexible and allowing for quick adjustments based on different material properties and printing needs. This technology effectively addresses issues such as monotonous food structures, poor palatability, and instability, making it particularly suitable for developing complex functional foods, including elderly food, dysphagia patient food, and personalized nutritional products. This significantly expands the practical application scope of 3D food printing technology. The coaxial extrusion principle and physical setup are illustrated in Figure 6.

### 3.3. Laser-Assisted 3D Printing

**Figure 7:**
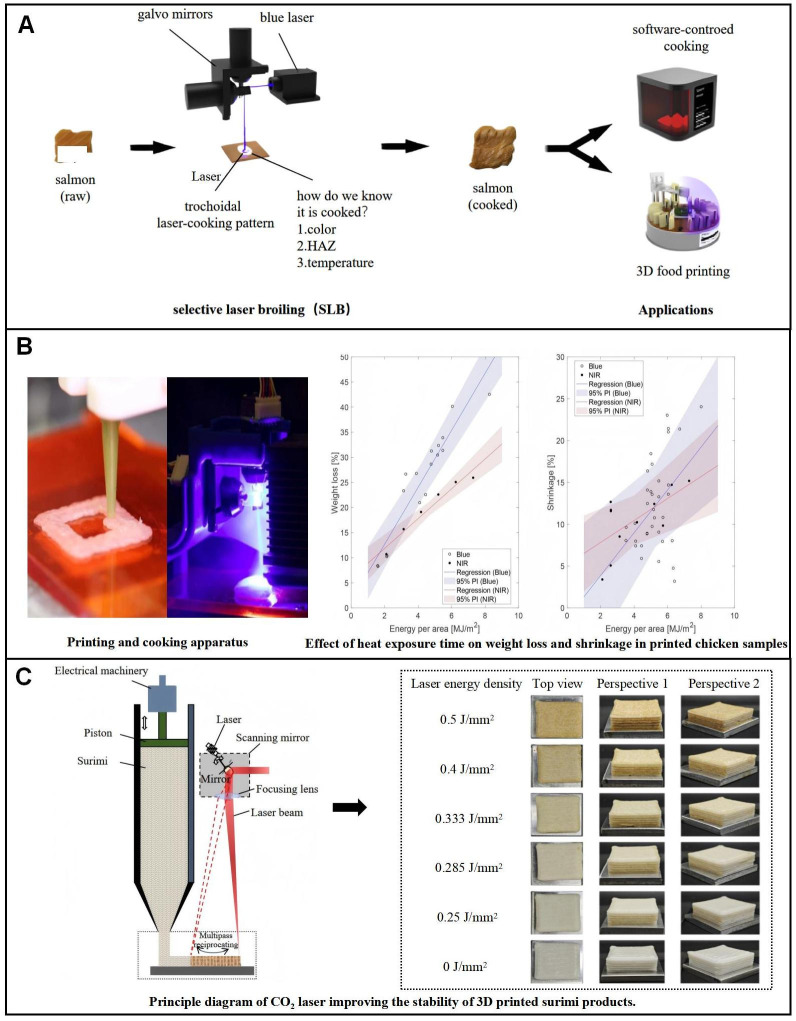
Laser printing application cases. (**A**) Selective Laser Grilling of Atlantic salmon [44]. (**B**) Three-dimensional printed chicken samples cooked precisely with multi-wavelength lasers [45]. (**C**) Study on the process of laser-assisted technology in surimi 3D printing preparation [21].

Laser-assisted 3D printing and selective laser sintering (SLS) represent two distinct technological approaches in the food manufacturing sector. Selective laser sintering utilizes high-energy lasers to selectively fuse or sinter food powder materials directly into desired shapes. However, it imposes more stringent requirements on food raw materials, typically necessitating specially formulated powder materials with appropriate melting and solidification characteristics. In contrast, laser-assisted 3D printing is a hybrid process that combines conventional extrusion-based 3D printing with laser cooking technology, utilizing lasers to provide precise thermal treatment while printing each layer of food material. This approach enables selective cooking, browning, or texture modification during the printing process while maintaining overall shape integrity. To validate the feasibility of laser cooking in food processing, Blutinger et al. employed lasers to cook Atlantic salmon, successfully measuring the food’s internal temperature and thermal penetration depth, as outlined in the workflow diagram shown in Figure 7A [44]. When using a 445 nm blue laser, the system achieved a thermal penetration depth of nearly 2 mm within the salmon tissue, inducing sufficient protein denaturation to ensure safe cooking temperatures (>62.8 °C). Furthermore, the study demonstrated that high-speed and repeated laser exposure could modulate surface color and texture properties. This research not only provides experimental validation for the application of laser-based cooking in meat processing but also highlights the potential of laser technology in precision food preparation. In another study, Blutinger et al. developed an innovative precision food cooking method that utilizes multi-wavelength lasers and is driven by customized software to control printing and cooking patterns [45]. The researchers employed a blue laser (λ = 445 nm), a near-infrared (NIR) laser (λ = 980 nm), and a mid-infrared (MIR) laser (λ = 10.6 μm) to cook chicken, comparing the effects of different laser wavelengths. Results demonstrated that infrared light induced more effective surface browning than blue light, while the NIR laser could penetrate packaging materials to simultaneously brown and cook the food. Additionally, laser-cooked chicken exhibited approximately 50% less cooking loss compared to traditional oven cooking (Figure 7B). This study marked the first integration of customized software into the cooking process, enhancing precision and enabling personalized food customization, thereby opening new market opportunities for the rapidly advancing food printing industry. Currently, most researchers modify meat and protein-based pastes using additives such as stabilizers, thickeners, salts, and microbial transglutaminase to obtain favorable fluid dynamic properties, enabling smooth extrusion while maintaining adequate structural strength. However, adding excessive additives merely to achieve printability contradicts the current food industry’s advocacy for “minimal additives” or even “zero additives” concepts.

**Figure 8:**
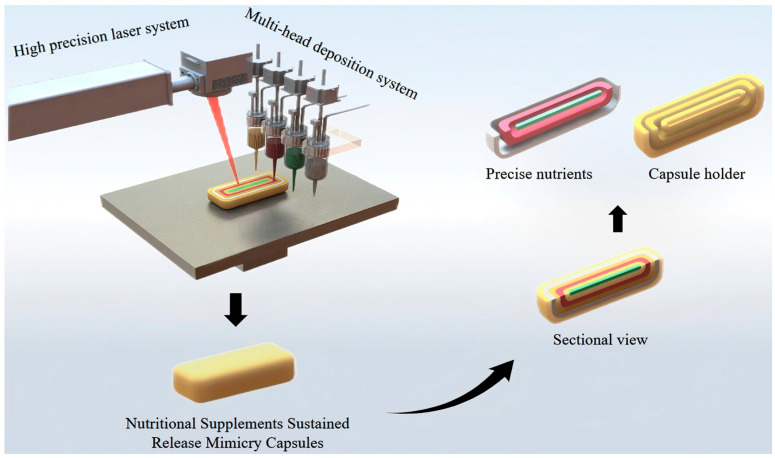
Multi-material 3D printing process composite molding process.

Our research team employed a 50 W CO_2_ laser to achieve rapid and precise localized heat-induced curing of 3D-printed surimi products (Figure 7C) [21]. By optimizing laser parameters to modulate energy density, we successfully fabricated surimi-based constructs with a thickness of 1.5 mm under conditions of 1 mm laser spot diameter, 500 mm/s scanning speed, and 0.25 mm scan spacing. This approach resulted in significant improvements in dimensional accuracy and gel strength compared to conventional methods, enhancing both product quality and processing stability while offering a more sustainable and efficient alternative to traditional manufacturing. In the future, we will focus on the development of multi-material laser-assisted 3D-printed nutrient supplement mimicry capsules with controlled release. By precisely regulating the release of different nutrients, these capsules can provide personalized and customized nutritional supplementation for specific populations, such as the elderly, athletes, and patients with chronic diseases. Utilizing a multi-compartment design, these capsules will enable the gradual release of various nutrients, ensuring a continuous supply of vitamins, minerals, amino acids, and other essential components over different time periods. This approach enhances bioavailability, reduces nutrient waste, and ensures sustained health management (Figure 8).

### 3.4. Microwave-Assisted 3D Printing

**Figure 9:**
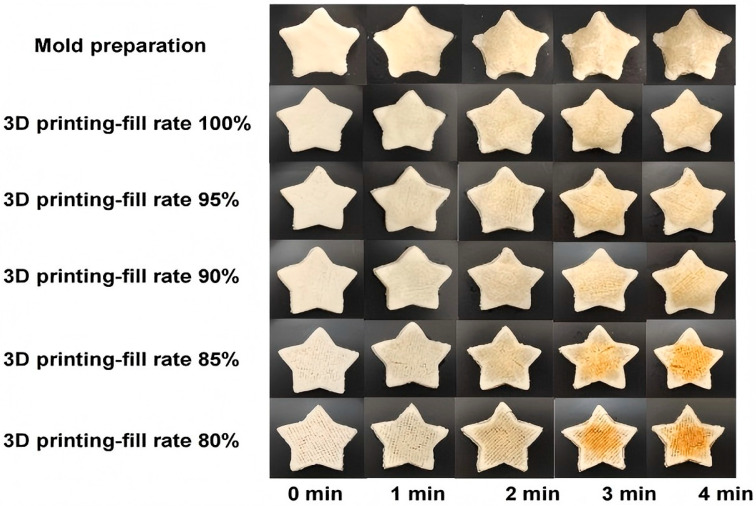
Comparative analysis of molded samples and 3D printing samples with different filling ratios after different microwave durations [49].

**Figure 10:**
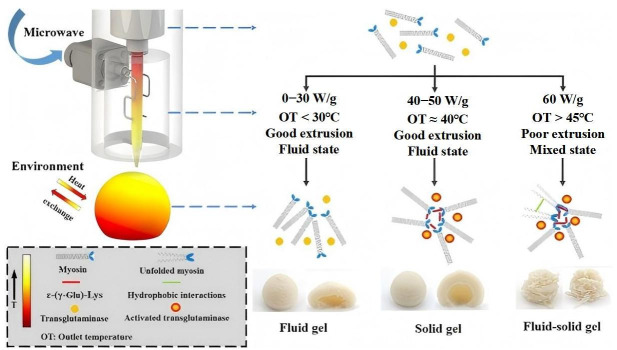
Proposed mechanism for the synergistic effect of MW3DP and TGase on the self-gelation [50].

Microwave 3D printing technology represents an innovative food manufacturing approach that integrates microwave heating with additive manufacturing. This method utilizes microwave radiation to achieve rapid heating and selective curing of materials during the printing process [46,47,48]. The lentinus protein (LP), a high-quality protein with a balanced amino acid profile, has shown promising potential in advanced food applications. Li et al. developed a microwave-assisted 3D-printed food system incorporating pea protein (LP) and potato starch [49]. Through formulation optimization (LP–potato starch–xanthan gum–water = 2:8:1:23), the study achieved optimal printability and structural integrity. The research compared 3D-printed samples with molded counterparts of varying infill densities under microwave processing. Post-treatment analysis revealed that 3D-printed specimens exhibited superior stability in shape, weight, and dimensional accuracy. Microwave heating duration was found to induce progressive top-surface hardening and interior softening while maintaining consistent hardness at the base (Figure 9). This research significantly advances the application potential of LP-based proteins in 3D food printing, offering optimized strategies for formulations, printing parameters, and microwave processing. It also demonstrates the feasibility of producing microwave-heated foods with desirable sensory attributes. To enhance the structural integrity of 3D-printed surimi products against self-gravity and post-processing disturbances, Zhao et al. investigated the synergistic effects of microwave-assisted 3D printing (MW3DP) and transglutaminase (TGase) on the autonomous gelation process of surimi during 3D printing, transitioning from a liquid to a solid gel state, as illustrated in Figure 10 [50]. Simulation and experimental results demonstrated that microwave power influenced the extrusion performance by altering the temperature distribution of surimi within the nozzle. The study also monitored the rheological properties of the extruded surimi and analyzed the post-printing gel characteristics, microstructure, and autonomous gelation mechanisms. When microwave power was below 60 W/g, the surimi exhibited shear-thinning behavior. After printing, under microwave powers of 40 and 50 W/g with TGase addition, the surimi products displayed enhanced shape fidelity and the formation of solid gels with large protein aggregates.

Moreover, compared to conventional heating methods, microwave heating demonstrates superior energy conversion efficiency and shorter heating duration. This not only reduces energy consumption but also better preserves the nutritional value of food products. Additionally, the relatively uniform temperature distribution during microwave heating effectively minimizes nutrient loss and food deterioration caused by localized overheating. However, microwave-assisted 3D printing technology still faces several challenges in its practical implementation. Primarily, controlling heating uniformity remains a significant concern. Due to variations in the dielectric properties of different food materials, localized overheating or non-uniform heating patterns may occur. Furthermore, increased system complexity presents another substantial challenge. The effective integration of microwave generators with 3D printing systems, while ensuring operational safety, imposes more stringent requirements on equipment design. With continuous technological advancement, microwave-assisted 3D printing is expected to play an increasingly crucial role in the food industry, driving food manufacturing technology toward intelligent and precise development.

### 3.5. Ultrasonic-Assisted 3D Printing

**Figure 11:**
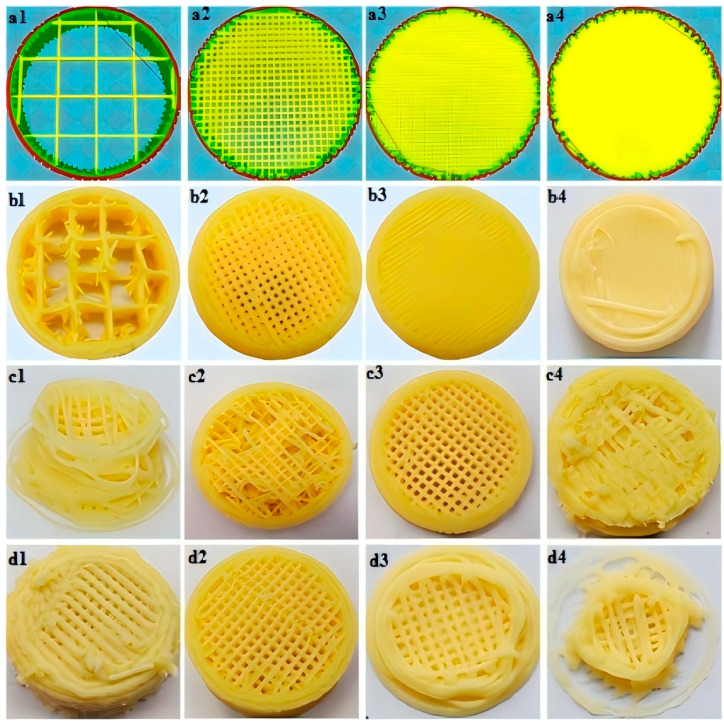
(**a**) Three-dimensional drawings of models designed considering different filling rates (a1 = 10%, a2 = 30%, a3 = 50%, a4 = 70%) as well as (**b**) photos of cylindrical cookie (b1–b4) (**c**) nozzle diameters (c1 = 0.4 mm, c2 = 0.6 mm, c3 = 0.8 mm, c4 = 1 mm) and (**d**) printing speeds (d1 = 25 mm/s, d2 = 50 mm/s, d3 = 75 mm/s, d4 = 100 mm/s) [54].

Ultrasound, laser, and microwave technologies all belong to physical intervention techniques, sharing the common characteristic of not relying on chemical additives, thus conforming to the “clean label” trend, while each possesses unique mechanisms and application advantages. Ultrasound-assisted technology utilizes high-frequency sound waves (≥20 kHz) to generate cavitation and mechanical shear, effectively altering rheological properties while protecting heat-sensitive nutrients [51,52]. Laser-assisted technology employs focused light energy to achieve precise heating, with its advantage lying in localized energy control precision [21]. Microwave-assisted technology relies on dielectric heating to achieve rapid volumetric solidification, combining uniform heating with high energy efficiency [50]. The differentiated functions of these three technologies in 3D printing provide diversified technical approaches for various food materials and structural requirements. During ultrasonic 3D printing, ultrasound energy generates intense cavitation effects, creating numerous microscopic bubbles within liquid food materials. The collapse of these bubbles results in extremely high localized temperatures and pressures. This unique physical phenomenon can alter the microstructure of food materials, such as inducing protein denaturation and starch gelatinization, thereby enabling precise control over food structure and texture [53]. Figure 11 investigates the effects of ultrasonic treatment and homogenization on the physicochemical properties of soybean residue dietary fiber in 3D-printed cookies. The results indicate that when soybean residue fiber is modified using a combination of 500 W and 15,000 rpm, the modified fiber exhibits excellent swelling capacity, water-holding capacity, and oil-holding capacity. Additionally, when the concentration of soybean residue fiber in the dough reaches 6%, the dough demonstrates optimal cohesiveness and printability. Incorporating modified soybean residue fiber into cookie dough not only enhances the utilization of the fiber but also increases the dietary fiber content of the cookies, thereby helping to mitigate the prevalence of obesity in modern populations [54].

Furthermore, ultrasonic treatment effectively inhibits microbial growth in food, reducing microbial content and extending the shelf life of printed food products [55]. Since ultrasonic treatment is a physical intervention method and does not involve the use of chemical additives, it aligns more closely with the principles of natural and healthy food processing. In recent years, ultrasonic treatment has garnered significant attention due to its notable emulsification and homogenization effects, as well as its antimicrobial properties. As shown in Table 2, the applications and performance impacts of ultrasound in 3D food printing are summarized.

### 3.6. Microfluidic 3D Printing

**Figure 12:**
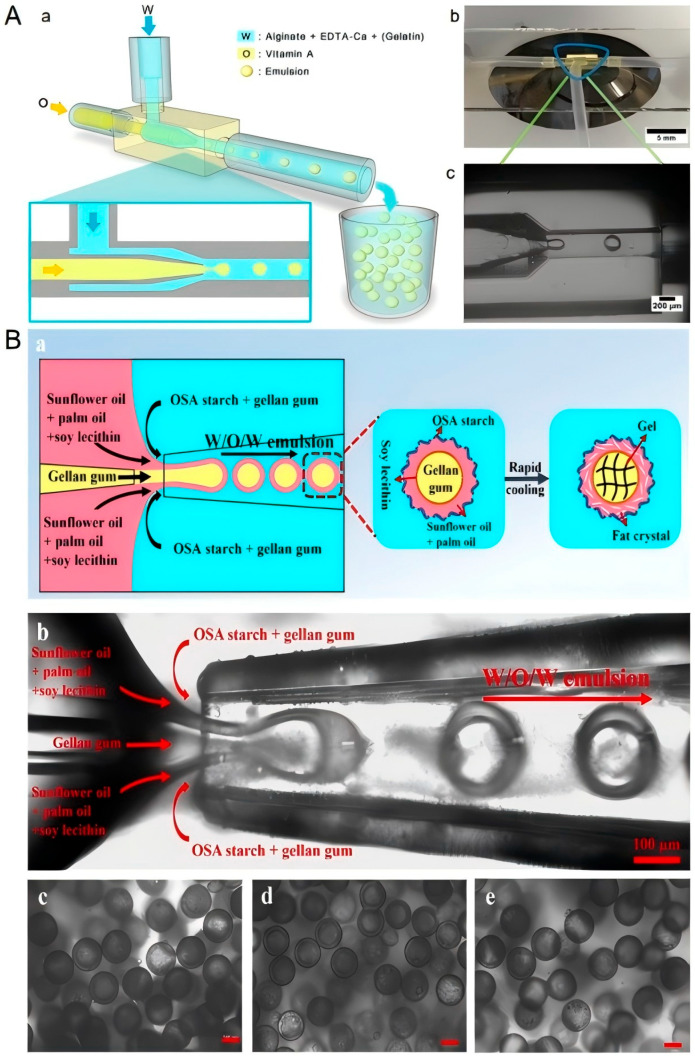
(**A**): (**a**) Schematic diagram of vitamin A microgel droplet generation. (**b**) Experimental platform of droplet formation. (**c**) Droplet generated by dripping under a 4× optical microscope [63]. (**B**): Schematic illustration (**a**) and optical microscopy (**b**) of the microfluidic fabrication of W/O/W mononuclear high internal phase double emulsions (MHIPDEs). Microstructure of MHIPDEs fabricated under different W1–O–W2 flow rate ratios: (**c**) 5-10-50, (**d**) 5-8-50, (**e**) 5-5-50, observed via inverted optical microscope in bright field. The scale bars are 100 μm [66].

Microfluidic 3D printing technology is a manufacturing method that combines microfluidics with 3D printing technology. This technology uses microscale channels and precise fluid control systems to achieve precise deposition of materials and microstructure regulation, so that functional products with complex internal structures can be precisely manufactured “bottom-up” [61]. At present, microfluidic 3D printing technology has made significant progress in the biomedical field, especially in drug delivery systems, tissue engineering, and medical device manufacturing [62]. Vitamin A is one of the essential fat-soluble vitamins for the human body, which is involved in maintaining normal vision, enhancing immunity, and promoting growth and development. Since the human body cannot synthesize vitamin A on its own, it must be obtained through food, with the intestine being its main absorption site. Zhang et al. studied the feasibility of 3D printing microfluidic encapsulation of vitamin A and found that two natural macromolecules, sodium alginate and gelatin, mixed with EDTA-Ca as a continuous phase, can form an oil-in-water (O/W) emulsion [63]. These emulsified droplets can be ingested by the human body and form microgel particles under the action of gastric acid, thereby preventing vitamin A from being degraded by gastric acid until it reaches the intestine for release. Figure 12A is a schematic diagram of the generation of vitamin A microgel droplets.

In the food field, microfluidic 3D printing technology is still in its infancy, but its application prospects are extremely broad [64,65]. The significant advantage of this technology lies in its excellent emulsification and dispersion properties. Through precisely designed microchannel structures, efficient droplet generation and control can be achieved to form uniform micron-sized droplets or particles. Figure 12B examines the role of microfluidic technology in the preparation of water-in-oil-in-water (W/O/W) single-core high internal phase double emulsion (MHIPDE) [66]. The study showed that MHIPDE with a moderate oil content (54 vol%) exhibited the best 3D printing performance and was able to print models with high resolution, realistic shapes, and smooth surfaces. The future application of microfluidic 3D printing technology in the food field is of great significance. Thanks to the extremely high accuracy and repeatability of the system, the technology can effectively ensure the uniformity and stability of printed food [67]. At the same time, through the precise material processing capabilities of microfluidic technology, functional foods with uniform distribution of nutrients and controllable release can be developed to make personalized nutrition more precise. However, micron-sized channels are prone to clogging, and strict control of material properties and process parameters is required. In addition, how to improve production efficiency while maintaining precise control is a key issue in the industrialization of this technology.

## 4. Potential Challenges of 3D Printing Food

### 4.1. Food Safety

Food safety concerns in 3D-printed foods span multiple stages, including raw material pre-treatment, the printing process, and post-processing. During the raw material pre-treatment stage, the preparation of ingredients involves processes such as grinding, mixing, and formulation, which are susceptible to microbial contamination. Particularly when preparing bioactive ingredients, it is essential to strictly control temperature and humidity to prevent the growth of pathogenic bacteria. Additionally, printing materials are at risk of microbial contamination when they come into contact with the printer’s extrusion pistons and pipelines or are exposed to air [68]. This necessitates strict control over the preparation and storage environments of raw materials.

Secondly, due to the complex structure of 3D printers, which include components such as nozzles, material tubes, and delivery pipelines, there are often hard-to-clean areas that conventional disinfection methods may not fully cover, creating potential breeding grounds for microorganisms. Severini et al. developed a smoothie using fruits and vegetables as 3D printing ink [69]. Microbiological analysis revealed that, despite rigorous cleaning of all printed products to reduce microbial contamination, contamination still occurred due to contact with the printer and prolonged exposure to air. Cross-contamination from residual materials is particularly critical when switching between different printing materials. Therefore, printer design should prioritize easy disassembly and cleaning, along with the establishment of standardized disinfection protocols and operating procedures.

In the post-processing stage, some 3D-printed foods require curing, baking, cooling, or other treatments. Inadequate temperature control during these processes can not only affect food texture but also lead to the formation of harmful substances or secondary microbial contamination. Thus, it is essential to establish scientifically validated post-processing parameters to ensure that foods achieve both ideal quality and meet safety standards.

### 4.2. Consumer Acceptance

Consumer acceptance of 3D-printed foods is influenced by multiple factors, making it a critical determinant for the successful marketization of this technology. Consumers have significant concerns about the perceived quality of 3D-printed foods, particularly regarding taste, appearance, and nutritional value. Incorporating consumer perspectives during the early development stages can help enhance market acceptance of novel food technologies [70]. The study results indicate that the innovativeness of 3D food printing technology significantly influences consumers and underscores that its successful market introduction hinges on practical applications and serving target groups. Brunner et al. demonstrated that consumers’ first impressions of the appearance of 3D-printed foods play a more critical role in shaping their opinions, while well-designed communication strategies can positively influence their attitudes toward such foods [71]. Additionally, Manstan and McSweeney highlighted that price significantly affects consumer acceptance, as the current production costs of 3D-printed foods are relatively high, creating a noticeable gap with public price expectations [72]. From the perspective of consumer demographics, Jayaprakash et al. found in their survey on the economic prospects and feasibility of 3D food printing technology that both experts and consumers support the application of 3D food printer (hardware–software) platforms [70]. These platforms can deliver functional value in terms of health, nutrition, and convenience, primarily due to the technology’s high maturity at the component level. Contextual acceptance of novel and unfamiliar foods (e.g., insects, cultured meat, plant-based meat alternatives) also influences consumer acceptance. Motoki and Park discovered that the anticipation of dining with friends or at food festivals plays a significant role in promoting the expected acceptance of insects, cultured meat, and 3D-printed foods [17]. Furthermore, increased positive emotions and reduced negative emotions may explain why these environmental factors enhance consumer acceptance of such foods. Cultural factors also play a crucial role in shaping consumer acceptance of novel foods. Lupton et al. noted that only a minority of participants expressed interest in or support for consuming or offering 3D-printed foods made from cultured meat or insects [73]. Many consumers expressed discomfort with the processing of cultured meat, perceiving it as “unnatural,” and raised concerns about its freshness, potential hazards, lack of flavor, and low nutritional value. Differences in dietary habits and cultural traditions across regions significantly influence consumer perceptions and acceptance of 3D-printed foods.

### 4.3. Relevant Regulations and Standard Systems

The healthy development of the 3D-printed food industry relies on a well-established regulatory and standards framework. Currently, the regulation of 3D-printed foods in various countries primarily draws on existing food safety regulatory frameworks. For instance, the “Technical Considerations for 3D-Printed Medical Products” issued by the U.S. Food and Drug Administration (FDA) provides partial reference points for 3D food printing [74]. In the European Union (EU), the Novel Food Regulation classifies 3D-printed foods as novel foods, requiring manufacturers to conduct safety assessments before market introduction [75].

In terms of raw material management, Baiano et al. noted that 3D-printed food materials must comply with food-grade standards and recommended the establishment of a dedicated raw material evaluation system [76]. Regarding equipment standards, Severini and Derossi emphasized that food-grade 3D printing equipment should meet requirements for food contact materials, including material standards for critical components such as printing nozzles and material storage chambers [69]. Meanwhile, Xiao et al. proposed the establishment of a traceability system for 3D-printed foods, encompassing requirements for recording key information such as raw material sources, printing parameters, and quality control. As the technology advances, related regulations and standards are also being continuously refined [19]. Tong and Xiao suggested the development of a comprehensive standard system covering raw materials, equipment, processes, and products to provide normative guidance for the healthy development of the industry [8].

## 5. Future Challenges and Opportunities

This article summarizes the research progress of 3D food printing technology in areas such as food safety, consumer acceptance, regulatory standards, and future development. Regarding the advancement of 3D-printed food technology, the following perspectives are proposed:

(1) The safety control system for 3D-printed foods still requires further improvement. Potential safety risks exist throughout the entire production process, from raw material pre-treatment to final product storage, necessitating the establishment of a comprehensive HACCP system. In particular, areas such as equipment sterilization, printing environment control, and shelf-life prediction demand in-depth research and enhancement.
(2) Consumer acceptance of 3D-printed foods remains relatively low, primarily due to concerns about product safety, nutritional value, and taste. Future efforts should focus on technological innovation to improve product quality, alongside strengthening public education to enhance consumer understanding and trust. Additionally, more application scenarios that meet consumer needs should be developed.
(3) The regulatory and standard systems need further refinement, especially in terms of raw material evaluation, equipment standards, process specifications, and product quality. A comprehensive standard system covering the entire industry chain should be established to provide normative guidance for industrial development. Simultaneously, international mutual recognition and harmonization of standards should be strengthened to promote global standardization.
(4) Future development directions will focus on new material development, technological innovation, and health applications, particularly in the fields of functional ingredient utilization, intelligent manufacturing technology integration, and personalized nutritional intervention. Additionally, implementing sustainable development strategies, including raw material recycling, energy conservation, emission reduction, and localized production, will become critical topics for industrial growth.
(5) The emergence of new 3D printing technologies presents fresh opportunities for 3D-printed foods. However, challenges remain in terms of equipment costs, operational complexity, and process standardization. Future efforts should strengthen industry–academia research collaboration to drive technological breakthroughs and promote the in-depth development of industrial applications.
(6) The transition from 3D printing to 4D printing transforms products from being entirely passive to becoming “smart foods” capable of actively sensing and adapting to environmental changes. In the future, research should focus on intelligent response mechanisms based on pH-responsive or temperature-sensitive materials, exploring how these materials can enable foods to undergo shape changes or structural transformations under specific conditions, thereby enhancing functionality and nutrient retention. Additionally, effectively preserving nutritional value and ensuring food safety will remain critical issues for further investigation.

## 6. Conclusions

Three-dimensional printing technology has achieved remarkable application results in the food industry, successfully producing a variety of innovative food products while also giving rise to numerous novel 3D food printing methods. This article systematically analyzes the main applications of 3D food printing technologies, including advanced techniques such as multi-axis 3D printing, coaxial 3D printing, selective laser sintering printing, microwave 3D printing, ultrasonic 3D printing, and microfluidic 3D printing. It delves into the characteristics of each technology and their potential uses in food manufacturing. Furthermore, the article highlights the challenges faced by 3D-printed foods, providing an in-depth analysis from three perspectives: food safety, consumer acceptance, and the establishment of relevant regulations and standards. The conclusion emphasizes that these three aspects are critical steps in advancing the development of 3D-printed foods and serve as important benchmarks for evaluating their commercial viability. In future research, priority should be given to the development and application of smart responsive materials, facilitating the transition from 3D printing to 4D printing technology. This will enable the creation of “smart foods” capable of autonomously adjusting their structures and properties in response to environmental conditions, offering consumers safer, smarter, and more personalized food choices, and driving innovation in the food manufacturing industry.

## Figures and Tables

**Table 1:** Comparison of different 3D food technologies.

	Extrusion 3D Printing	Inkjet 3D Printing	Hydrogel Molding Extrusion Printing	Adhesive Spray Molding Technology Printing	Selective Laser Sintering	Selective Hot Air Sintering
Schematic diagram	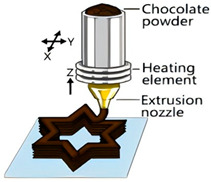	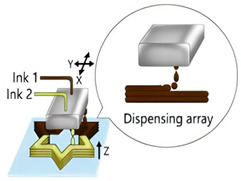	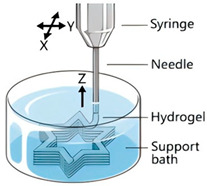	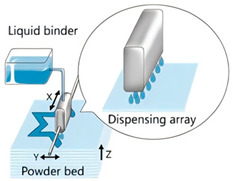	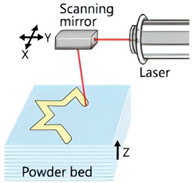	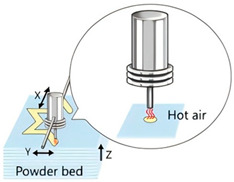
Advantages	Applicable to a variety of materials, simple equipment.	High resolution for fine patterns and colorful food decorations.	Capable of printing complex hydrogel structures.	No heating required, fast printing speed.	High precision, suitable for complex geometries.	No laser source required, low equipment cost.
Available materials	Chocolate, cheese, fruit puree, meat puree, etc.	Syrups, sauces, etc.	Hydrogel, trehalose, pectin, etc.	Starch, powdered sugar, flour, and other food powders.	Sugar powder, chocolate, fat, and other food powders	Flour, powdered sugar, and other food powders.
Application scope	Personalized food customization, catering creativity, etc.	Food surface decoration, personalized patterns, customized cakes, etc.	Soft foods (jelly, gummies, etc.)	Food powder molding, biscuits, bread, etc.	High-precision creative food, exquisite candies, personalized food, etc.	Low-sugar and low-fat customized food.
Printing limitations	Slow printing speed; cannot handle low-liquid ingredients.	Slow printing speed; Only suitable for liquid or colloidal state materials.	Slow printing speed; high temperature control requirements, limited applicability	High requirements for glue spray control; high requirements for powder fluidity, particle size, etc.	High powder quality and particle size requirements; high equipment costs.	Strict temperature control requirements; accuracy is not as good as laser sintering, but suitable for simple food structures.
Print products	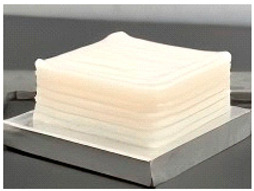	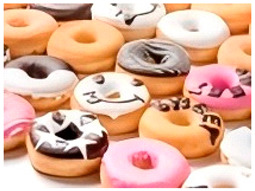	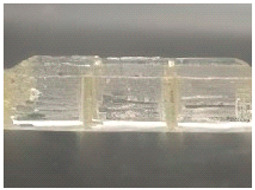	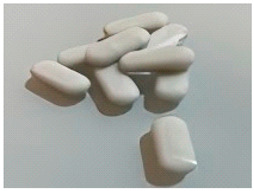	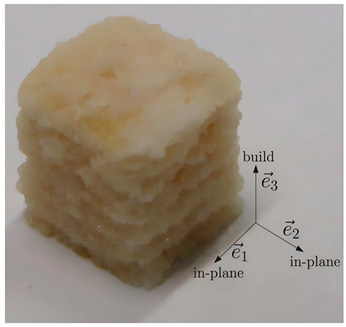	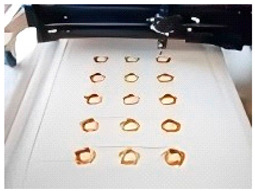
References	[20,21]	[20,22]	[20,23]	[20,24]	[20,25]	[20,26]

**Table 2:** Impact of ultrasonic waves on 3D food printing applications and performance.

Printing Materials	Print Parameters	Conditions	Ultrasonic Application Mode	Significant Findings	Reference
Wheat Starch-Papaya	Temperature: 25 °C, nozzle diameter: 1.0 mm, layer height: 1.0 mm, printing speed: 15 mm/s.	Ultrasonic: 80 W; 30 min, microwave: 80 W; 4 min.	Indirect (pre-treatment)	Improve the stability and accuracy of 3D printed samples.	[56]
Silver carp surimi	Temperature: 25 °C, nozzle diameter: 1.5 mm, layer height: 1.5 mm, printing speed: 15 mm/s.	Frequency: 45, 80, and 100 kHz.	Indirect (pre-treatment)	Reduce damage to the myogenic protein structure; improve the printing accuracy of surimi gel; improve the hardness, elasticity, and chewiness of printed products.	[57]
Wheat bran	Temperature: 20 °C, nozzle diameter: 1.0 mm, layer height: 0.4 mm, printing speed: 25 mm/s.	Power: 400 W, time: 2 min.	Indirect (pre-treatment)	Reduce polyphenol oxidase activity and effectively stop the browning process; increase the viscosity of dough and enhance the accuracy of printed snack shapes.	[58]
Pectin and pea protein	Temperature: 25 °C, nozzle diameter: 0.84 mm, layer height: 0.84 mm, printing speed: 15 mm/s, filling density 60%.	Power: 390 W, frequency: 29 kHz, time: 10 min.	Indirect (pre-treatment)	Incorporating pectin after ultrasonic treatment can improve the texture and rheological properties of the pea protein matrix; improve the mechanical strength and stability of the printed product.	[59]
Popcorn	Voltage: 5–16 V, printing accuracy: 0.16 mm, vertical speed: 25 cm/s, horizontal speed: 12.5 cm/s.	Ultrasonic phased array: A phased array composed of 16 × 16 40 kHz ultrasonic transducers (model MA40S4S).	Indirect (pre-treatment)	Developed a non-contact 3D printing method based on ultrasonic phased array. It is highly precise, sensitive, non-contact, and has a wide range of applications; it enables rapid movement of suspension points.	[60]

## Data Availability

No new data were created or analyzed in this study.
